# Awareness of glaucoma among adult patients attending hawassa university comprehensive specialized hospital ophthalmic outpatient department, Sidama, Ethiopia, August 2022

**DOI:** 10.1186/s12886-024-03517-3

**Published:** 2024-06-10

**Authors:** Balcha Negese Kebede, Seid Mohammed Seid, Biruktayit Kefyalew

**Affiliations:** Department of Ophthalmology and Optometry, Hawassa University college of Medicine and health Sciences, Hawassa, Ethiopia

**Keywords:** Awareness, Glaucoma, Hawassa

## Abstract

**Background:**

Due to the asymptomatic nature of the disease and lack of awareness, most glaucoma patients present for eye examination late, after significant damage of optic nerve occur. Being aware of glaucoma is important for timely diagnosis of the disease and preventing blindness from it.

**Objective:**

The aim of this study was to assess glaucoma awareness and associated factors among adult patients aged 35 and over attending the eye outpatient department.

**Methods:**

Hospital based cross-sectional study was conducted on 284 adult patients aged 35 and over attending ophthalmic outpatient department from July to August 2022 using systematic random sampling. An interviewer-administered questionnaire was used to collect data. The data were checked for completeness, and then entered to SPSS version 22 software. Descriptive and binary logistic regression analyses were performed. Independent variables with *p*-value ≤ 0.05 in multivariate logistic regression were considered as statistically significant.

**Results:**

About 284 study participants, of whom 57.75% were male, participated in this study with a response rate of 94.1%. The mean age of the study participants was 53.58 years. Only 39.09%(95% CI: 36.53–41.65) were aware of glaucoma. Age groups 46–50 [AOR; 1.83: 1.18, 2.56] and 51–64 [AOR; 3.21: 2.03, 4.39], having college education or above [AOR; 3.1: 2.20, 6.64], family member with glaucoma [AOR; 5.86:3.25, 8.0], income 6500 ETB [AOR; 2.9: 1.97, 5.00] and previous eye examination [AOR; 2.15: 1.46, 4.05] were factors significantly associated with awareness of glaucoma. The main sources of information were news media, family members with glaucoma and health workers.

**Conclusion:**

More than half (60.91%) of adult ophthalmic patients attending HUCSH were unaware of glaucoma and need eye health education concerning glaucoma.

**Supplementary Information:**

The online version contains supplementary material available at 10.1186/s12886-024-03517-3.

## Background

Glaucoma refers to a group of ocular disorders characterized by progressive optic nerve atrophy for which the major and treatable factor is intraocular pressure [1, 2]. It is the leading cause of irreversible blindness, although it is the third leading cause of blindness worldwide following cataract and trachoma [3]. Glaucoma accounts for 15% of blindness globally and about 600,000 people became blind from the disease each year [4]. In 2010, it affected almost 60.5 million people worldwide [4]. About 15% of people blind due to glaucoma were living in Africa, and the situation is worse in the sub-Saharan Africa [4].

Intraocular pressure above 21 mmHg is the most common and treatable risk factor for glaucoma [5]. Lowering IOP with anti-glaucoma medication and surgery is a key to hinder disease progression and blindness from it [6].

Early detection is crucial to tackle blindness due to glaucoma. Awareness of the disease nature has a great role in increasing regular eye check-ups and early diagnosis. From literatures reviewed, glaucoma awareness among adult patients ranges from 2.4% [8] to 74.0% [16]. Since glaucoma is asymptomatic in nature and affects central vision after advanced stage of the disease, many patients visit health facilities late after visual field and optic nerve have been significantly damaged. This may be due to a lack of its awareness. But, there is limited evidence on the level of patient awareness of glaucoma in the study area. Therefore, assessing awareness of glaucoma among patients attending Hawassa University comprehensive specialized hospital (HCSH) ophthalmic outpatient department (OPD) is important; as it provides baseline information on the level of awareness of glaucoma that could help policy makers for planning eye care services and researchers to conduct further studies.

## Methods and materials

### Study design, study area and period

A cross -sectional study was conducted at HUCSH which is located in Hawassa, the capital city of Sidama regional state, of Ethiopia. It found at 275 Km south of Addis Ababa. The study was conducted from July 01 to August 30, 2022 on adults who presented at HUCSH Ophthalmic OPD.

### Study population

Adult patients ≥ 35 years age were selected from ophthalmic patients presented at HUCSH Ophthalmic OPD during the data collection period.

### Inclusion and exclusion criteria

Adult patients aged 35 years and over attending ophthalmic OPD during the data collection period were included to study. However; patients on glaucoma follow-up, assumed to be obviously aware of glaucoma as well as, those mentally incompetent and unable to communicate were excluded.

### Sample size determination

Sample size was determined using a single proportion estimate formula, by taking the proportion(P) from the study conducted at St. Paul’s Hospital, a tertiary care center in Addis Ababa, which was 44.0% [7]. By taking 95% confidence interval, 5% marginal errors and 10% non-response rate, the sample size was calculated as follows:

$$n = \frac{{{{\left( {{Z_{{\raise0.7ex\hbox{$\alpha $} \!\mathord{\left/{\vphantom {\alpha 2}}\right.\kern-\nulldelimiterspace}\!\lower0.7ex\hbox{$2$}}}}} \right)}^2}P(1 - P)}}{{{d^2}}}$$

Where n – sample size, Z = confidence level = 1.96, P = proportion = 0.44% and d = margin of error = 0.05. n = (1.96)^2^(0.44) (0.56)/ (0.05)^2^=378.

Since our study population was ophthalmic patients who were small segment of adult population, which is less than 10,000 patients, attend the hospital during study period. Estimated number of patients attending the hospital during study period based on the pattern of patient flow to the hospital was around 1000. So that minimum sample size less than 378 could represent them; Final sample size calculated using sample size correction formula as follows.

$nf$_=_$\frac{n}{1+n/N}$ = $\frac{378}{1+378/1000}$ =274, by adding non-respondent rate 10% which is 28, we get sample size to be 302.

### Sampling technique

A systematic random sampling technique was used to select 302 study participants (n). The projected number of patients attending adult ophthalmic OPD during the collection was 1000(N). Therefore, the sampling interval ‘K’ calculated as (k = N/*n* = 1000/302 ≈ 3). The first study participant was selected from the first three attendants at the beginning day of data collection, by the lottery method, and then every 3rd patient in their order of arrival to the OPD were interviewed.

### Study variables

Awareness of glaucoma was the dependent variable, while age, sex, religion, marital status, address, educational level, occupation, income, history of eye examination and diabetic mellitus were the in dependent variables.

### Operational definition

The study subjects were considered aware if he or she was able to answer at least one of the questions used to define glaucoma; in addition to a positive response (‘Yes’) for question ‘have you ever heard of glaucoma [8]?.

### Data collection procedure and quality control

The structured questionnaire developed was pretested for its reliability and validity on 5% of the total sample size on adult ophthalmic OPD patients of Adare general hospital. The questionnaire was assessed for its clarity, completeness, reliability and necessary amendments were made. Validity of the questionnaire was checked by interviewing the same individuals three times by different interviewer using the same questionnaire. Cronbach’s alpha calculated for pretest 5% data using SPSS software was 0.83. Since Cronbach’s α > 0.7 indicate reliability of data extraction tool, the questionnaire was accepted as reliable. The validated questionnaire was translated to three local languages by language expert for data collection purpose, and then retranslated to English after data collection.

### Data processing and analysis

The collected data were checked for completeness and then entered into SPSS 22. Descriptive analysis was performed using the software; the findings were presented in tables. Binary logistic regression was also performed using SPSS 22 to identify independent variables associated with awareness of glaucoma. Confidence interval of 95% with AOR was used to indicate strength of association. Independent variables with *P*-value of 0.05 or less in multivariate analysis were considered as statistically significantly associated with glaucoma awareness.

## Result

### Socio-demographic characteristics of study participants

A total of 284 adult patients aged 35 and above participated in the study, with a response rate of 94%. The mean age of study participants was 53.58 ± 4.67SD years. Majority of the study participants were male (57.75%). Around 78.17% of them had formal education while 21.83% of them were unable to read and write (Table 1).

**Fig. 1:**
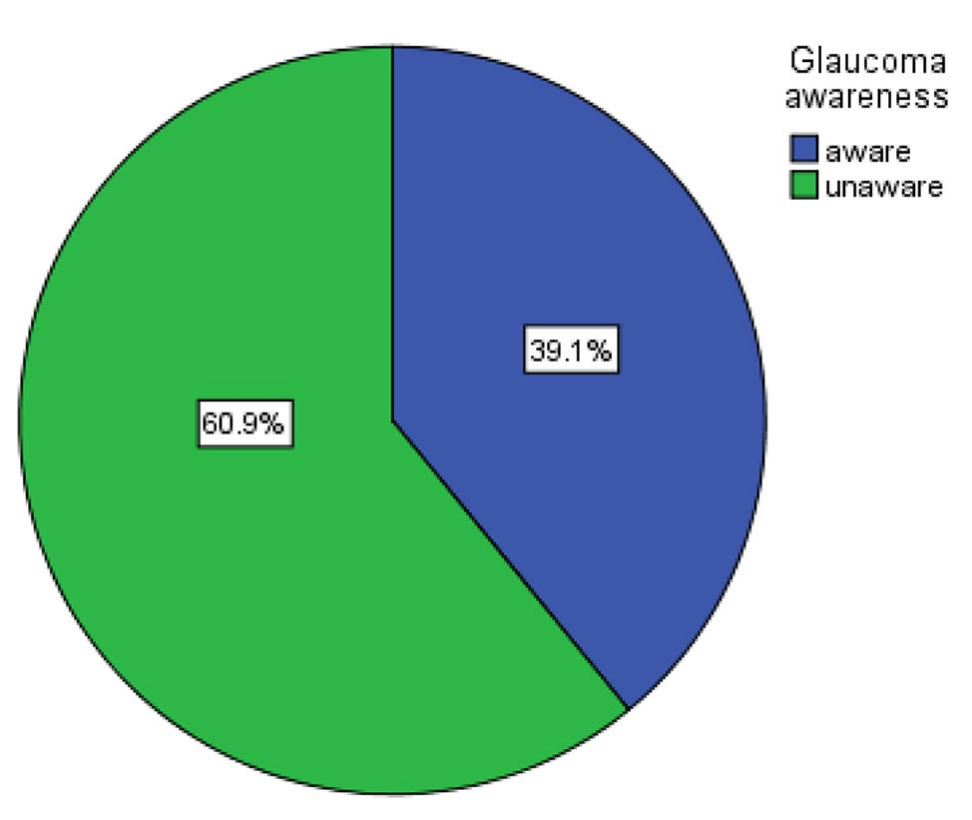
Proportion of glaucoma awareness among adults patients attending HUCSH Ophthalmic OPD.

Their level of awareness about glaucoma determined using validated and reliable questionnaire with Cronbach’s alpha (α) 0.83. From 284 patients interviewed, about 124 study participants heard about glaucoma but only 111 (39.09%) (95% CI: 35.53–42.65) were aware of it (Fig. 1).

**Table 1:** Socio-demographic factors of study participants (*n* = 284)

Variable	Category	Frequency(*n*)	Percent (%)
Age	35–45	95	33.45
	46–50	48	16.9
	51–64	75	26.41
	65 and above	66	23.24
Sex	Male	164	57.75
	Female	120	42.25
Educational	Unable to read and write	49	17.25
Status	Read and write	62	21.83
	1–8 grade	51	17.96
	9–12 grade	69	24.30
	College and above	53	18.66
Religion	Orthodox	70	24.65
	Muslims	92	32.39
	Protestant	84	29.58
	Others	38	13.38
Residency	Urban	130	45.77
	Rural	154	54.23
Marital status	Single	27	9.51
	Married	157	55.28
	Divorced	49	17.25
	Widowed	51	17.96
Income in ETB	0-4000	89	31.34
	4001–5500	74	26.06
	5501–6500	67	23.59
	≥ 6500	54	19.01

Out of 111 participants who were aware of glaucoma, 66(59.5%) explained glaucoma as high eye pressure damaging the eye, 18 (16.21%) explained it as eye nerve damage, 14(12.61%) defined as it cause irreversible blindness and 13 (11.71%) explained glaucoma as visual field loss. Out of the 114 study subjects who had eye examinations, 62 (54.34%) were aware of glaucoma. The main source of information was news media 49/124(39.51%), followed by having family members with glaucoma 45/124(36.29%) and health workers 25/124 (20.16%) (Table 2).

**Table 2:** Glaucoma awareness related questions

Glaucoma awareness related questions	Frequency (percent %)
Have you ever heard about glaucoma? (*n* = 284)	
Yes	124(43.66)
NO	160(56.34)
How do you define glaucoma? (*n* = 124)	
High intra ocular pressure	66(53.23)
Damage optic nerve	18(14.52)
Causes irreversible blindness	14(11.29)
Causes visual defect	13(10.48)
I don’t know	13(10.48)
From where did you hear about glaucoma? (*n* = 124)	
News media	49(39.52)
From family member with glaucoma	45(36.29)
Health care workers	25(20.16)
Others	5(4.03)

On multi-variate logistic regression analysis, age groups 46–50 and 51–64 with AOR of 1.83 (95% CI: 1.18–2.56) and 3.21(95% CI: 2.03–4.39) respectively, having college education and above [AOR = 3.1; 95% CI: 2.20–6.64], Family member with glaucoma [AOR = 5.86; 95% CI: 3.25-8.0], income 6500 ETB [AOR = 2.9; 95% CI: 1.97-5.00] and previous eye examination [AOR = 2.15; 95% CI: 1.46–4.05] were factors significantly associated with awareness of glaucoma(Table 3).

**Table 3:** Factors associated with awareness of glaucoma (*n* = 284)

Variables	Categories	Aware	Unaware	OR(95% CI)	AOR (95% CI)
Age	35–45	29	66	1.27(1.01–2.93)	1.15(0.73–3.47)
	46–50	21	27	2.24(1.56–3.79)	1.83(1.18–2.56)*
	51–64	44	31	4.1(2.51–5.67)	3.21(2.03–4.39)*
	≥ 65	17	49	1.00	1.00
Sex	Male	52	112	1.00	1.00
	Female	59	62	2.05(1.73–3.06)	1.16(0.52–3.6)
Educational status	Unable to read and write	12	37	1.00	1.00
	Read and write	18	44	1.26(0.33–2.76)	1.03(0.21–2.55)
	1–8 grade	20	31	1.99(0.17–3.9)	1.12(0.36–4.12)
	9–12 grade	30	39	2.37(1.24–3.52)*	1.72(0.94-6.0)
	College and above	31	22	4.80(2.90–7.72)*	3.1(2.20–6.64)*
Religion	Orthodox	28	42	1.02(0.51–4.83)	1.06(0.49–5.10)
	Muslims	35	57	0.94(0.48–3.44)	0.89(0.6–3.2)
	Protestant	33	51	0.99(0.65–3.59)	0.92(0.56–4.71)
	Others	15	23	1.00	1.00
Residency	Urban	69	61	3.02(2.03–4.11)*	1.75(0.96–5.26)
	Rural	42	112	1.00	1.00
Marital status	Single	10	17	1.00	1.00
	Married	62	95	1.11(0.63–7.45)	0.87(0.34–5.90)
	Divorced	19	30	1.08(0.32–2.41)	0.92(0.43–3.12)
	Widowed	20	31	1.10(0.28–2.34)	0.77(0.62–2.74)
Income in ETB	0-4000	25	64	1.00	1.00
	4001–5500	23	51	1.15(0.85–3.16)	0.86(0.64–3.15)
	5501–6500	31	36	1.56(1.00-3.14)*	1.13(0.78–4.26)
	> 6500	29	22	3.37(2.04–5.06)*	2.9(1.97-5.00)**
Family history with glaucoma	Yes	40	5	6.13(3.59–8.14)*	5.86(3.25-8.0)
	Don’t know	10	124	0.06(0.34–1.16)	0.12(0.08–1.35)
	No	64	49	1.00	1.00
Eye examination before	Yes	61	53	2.76(1.95-6.0)	2.15(1.46–4.07)*
	No	50	120	1.00	1.00
History of diabetic Mellitus	Yes	6	12	1.03(0.43–2.21)	0.40(0.23–1.82)
	Not screened	61	70	1.80(0.83–3.32)	1.45(0.95–3.11)
	No	44	91	1.00	1.00

## Discussion

The awareness of glaucoma among adults aged 35 and above in current study was 39.09%. This proportion was higher than reports from previous study conducted in Agaro town 2.40% [8]. The reason for the disparity was that participants in Agaro town were those with low economic status seeking charity service. Income was significantly associated with glaucoma awareness in this study. Similarly, it was higher than reports from central, North and South India 27% [9], 26.10% [10], 27.2% [11] respectively. More proportion of study participants in this study had family members with glaucoma compared to previous studies which can be considered as reason for the difference, because having family member with glaucoma was significantly associated with glaucoma awareness. It was also the second commonest main source information about the disease. This indicate that increasing means of diagnosing people with glaucoma through conducting glaucoma screening campaign and making eye care service easily accessible is one part of health education about glaucoma for family members of individuals with glaucoma. It was also, higher than finding from a study at Menelik II Hospital, Addis Ababa Ethiopia which reported 28.44% [12]. In the same manner, it was higher than 27.2% reported from a study in Ghana [13]. The difference was due to slight difference in age of study participants between the current and previous studies.

However, the finding about level of glaucoma awareness in the present study is lower than the findings from studies in Sydney (72%)^14^, Nepal (60.6%) 15, Ghana (74%) [16] Zambia (71.5%) [17] and St. Paul’s Hospital, a tertiary care center in Addis Ababa, which was (44.0%) [18]. The reason disparity was that socio economic status deference of study participants of current and previous studies. All of those countries have more growth domestic income compared to Ethiopia. Similarly, socio economic status of Addis Ababa is better than other part of the country because it is the capital city of the country. In addition, previous studies included all adults above 18 years which can be a reason for the difference.

However, the level of glaucoma awareness in this study was similar to the finding a study conducted in Ghana Abokob which was (39.33%) [19].

### Factors associated with glaucoma

In this study, age from 46 to 64 years, positive history of eye examination, having income above 6500 ETB and having family member with glaucoma were significantly associated with glaucoma awareness.

Being in the age groups of 46–50 and 51–64 years were almost twice and three times more likely to be aware of glaucoma compared to age group of 35-45years respectively. This is similar to the findings in a study from USA where participants in age group 50–64 were 1.4 times more likely to be aware of glaucoma [14]; Also, a study from Sydney, Australia observed that participants aged above 40 were twice more likely to be aware of glaucoma [20].

Those who has eye examination were almost twice more likely to be aware of glaucoma compared to those have not. This is similar to the observation in a study from Ghana where those who had previous eye examination were 1.41 more likely to be aware of glaucoma [13].

Individuals who attended college and above were 3.1 times more likely to be aware of glaucoma. This is similar to the findings in studies from central India [9], Nepal [15] and Ghana Komfo Ankoye hospital [16]. However, in another study from Ghana [13], all levels of education were inversely proportional with awareness.

Individuals who have family member with glaucoma were approximately six times more likely to be aware of glaucoma. This is similar to findings in study from Sydney, Australia where individuals with first relatives who has had glaucoma were15.7 times more likely to be aware of glaucoma [20].

## Conclusion and recommendation

The awareness of glaucoma in this study is below 50%, which is relatively low and it is better to raise glaucoma awareness in the study area through glaucoma eye health education both at hospital and community level. Ophthalmology Society of Ethiopia better to provide information about glaucoma regularly using different mass media. Different donors and government are enjoined better to support eye health education. Educational level and income were the two modifiable factors associated with glaucoma awareness in the study. Therefore promoting quality education and increasing income of the community are important to increase eye health literacy including glaucoma.

### Electronic supplementary material

Below is the link to the electronic supplementary material.

Supplementary Material 1

Supplementary Material 2

Supplementary Material 3

## Data Availability

Data from which analysis done available on request from Balcha Negese/balchanege@gmail.com.

## Abbreviations

HUCSH: Hawassa University comprehensive specialized hospital
IOP: Intraocular pressure
OPD: Outpatient department
MSc: Master of Science
