# Supplementary material for: Awareness of glaucoma among adult patients attending hawassa university comprehensive specialized hospital ophthalmic outpatient department, Sidama, Ethiopia, August 2022
Source: BMC Ophthalmol. 2024 Jun 10;24:243. doi: 10.1186/s12886-024-03517-3 (PMC11163766; doi:10.1186/s12886-024-03517-3)
Supplement: Supplementary file 1 — Supplementary Material 1 [file 12886_2024_3517_MOESM1_ESM.docx]

Annexes

| Part I.Sociodemagraphic factors of the study participant | | |
| --- | --- | --- |
| Age | |  |
| Sex | | [1.] Male [2].Female |
| Educational status | | [1].Unable to read and write [2]. Read and write  [3]. 1-8 grade [4].9-12 grade [5]. College and above |
| Religion | | [1].Orthodox [2]. Muslims [3]. Protestant [4].Others |
| Residency | | [1].Urban [2].Rural |
| Marital status | | [1].Single [2].married [3].divorsed. [4].widowed |
| Income in Ethiopian birr | | [1].0-4000 [2].4001-5500 [3].5501-6500 [4]. >6500 |
| Part II.Awereness related questions | | |
| Have you ever heard glaucoma? | | 1.Yes 2.No |
| From where did you first hear about glaucoma? | | 1.news media 2.posters 3.school 4.health worker  5.relative with Glaucoma 6.Others |
| How do you explain glaucoma? | | 1.associated with high intraocular pressure  2.Cuase damage to eye nerve(optic nerve)  3.Asymptomatic 4. Blind the eye  5.cause visual field defect  6. I don’t know |
| Part III Medical and ophthalmic history | | |
| Do you have family member with glaucoma | 1. Yes 2.no 3. I don’t know | |
| Have you Diabetic mellitus? | 1.Yes 2.no 3.Idont know or not screened | |
| Have you hypertension? | 1.Yes 2.no 3.I don’t know or not screened | |
