# Supplementary material for: Awareness of glaucoma among adult patients attending hawassa university comprehensive specialized hospital ophthalmic outpatient department, Sidama, Ethiopia, August 2022
Source: BMC Ophthalmol. 2024 Jun 10;24:243. doi: 10.1186/s12886-024-03517-3 (PMC11163766; doi:10.1186/s12886-024-03517-3)
Supplement: Supplementary file 2 — Supplementary Material 2 [file 12886_2024_3517_MOESM2_ESM.docx]

**English version of data extraction format**

Pre tested semi-structured questioners with data extraction form for glaucoma awareness and associated factors among adult ophthalmic patients attending Hawassa university comprehensive specialized hospital

**Introduction**

Good morning/afternoon, my name is -------------------------------- I am a student in Hawassa University. I am a member of a research group being conducted in Hawassa University comprehensive specialized hospital. I am studying the level of glaucoma awareness and associated factors in adult ophthalmic clients attending Hawassa university comprehensive specialized hospital by asking questions. Your truth full answers for all of our questions are important to know level of glaucoma awareness and associated factors. Your answers will be confidential and secret. If you decide that, you do not want to participate in the study now or at any time in the future; you have full right to not participate. The participation is voluntary; refusing to participate will not have any associated risk for you. But we appreciate you if you try to participate and give us 15 minutes to complete the questionnaire. Thank you. Next, I will read a consent, which assures your interest to participate.

Do I have your permission to continue? If no, thank you and go to next study subject

If yes thank you and continue -------------

Data collector

Name --------------------- signature---------------------- date------------------

Checked by supervisor

Name ------------------------------ signature------------------- date-------

**Data collection tool for’ Glaucoma awareness and associated factors among ophthalmic clients attending HUCSH**

Encircle number with appropriate options (answer); select all possible answers if your answer is more than one.

| s/n | Research questions | Possible answers | skip |
| --- | --- | --- | --- |
|  | Socio- demographic factors |  |  |
| 1 | ID |  |  |
| 2 | Sex | 1. Male 2. Female |  |
| 3 | Age in years | ______________ |  |
| 4 | Residency | 1. Urban 2. Rural |  |
| 5 | Educational status | 1.unable to read and write  2.Able to read and write  3.primary (1-8)  4.secondary (9-12)  5. college and above |  |
| 6. | Religion | 1. Orthodox |  |
|  |  | 2.Muslims |  |
|  |  | 3.Protestant |  |
|  |  | 4.catholic |  |
|  |  | 5. others, specify____________ |  |
| 7 | Marital status | 1.single  2.married  3.Divorsed  4.Widowed |  |
| 8 | Average monthly income in ETB | _________________ |  |
|  | Glaucoma awareness question |  |  |
| 9 | Have you ever heard about glaucoma? | 1. Yes 2. No | If no, pass to Q12 |
| 10 | If yes, how you define or explain glaucoma | 1.Increased intraocular pressure damaging the eye  2. It damages optic nerve  3. It causes irreversible blindness  4.It causes visual field defect  5.I don’t know |  |
| 11 | From where did you heard about glaucoma? | 1.news media  2. health worker  3. family member with glaucoma |  |
|  |  | 4.school |  |
|  |  | 5.other |  |
| 12 | Have you family member with glaucoma? | 1. Yes 2. No |  |
| 13 | Have you ever had eye examination before? | 1. Yes 2. No |  |
| 14 | Have you diagnosed of Diabetic mellitus | 1. Yes 2. No |  |
| 15 | If yes, have screened for diabetic eye disease? | 1. Yes 2. No |  |
