# Supplementary material for: Awareness of glaucoma among adult patients attending hawassa university comprehensive specialized hospital ophthalmic outpatient department, Sidama, Ethiopia, August 2022
Source: BMC Ophthalmol. 2024 Jun 10;24:243. doi: 10.1186/s12886-024-03517-3 (PMC11163766; doi:10.1186/s12886-024-03517-3)
Supplement: Supplementary file 3 — Supplementary Material 3 [file 12886_2024_3517_MOESM3_ESM.docx]

**Respondent consent form for interview**

*Dear* Respondent

You are participating in survey to be undertaken to detect level of glaucoma awareness and its associated factors. Glaucoma is blinding ocular disease commonly affecting an individual above 35 years. Blindness due to glaucoma is irreversible, so that awareness of the disease nature has great role in preventing sight loss from it. This makes it important to identify the level of glaucoma awareness and associated factors. So that you have a better chance to get import information about glaucoma. Your response will be collected for research and program planning purposes by Hawassa University, college of Medicine and Health Science Department of Ophthalmology and Optometry.

With your permission, we would like to:

1. Conduct interview with you
2. Collect demographic and glaucoma awareness related information from you.

If we find that you as unaware of glaucoma we will give you eye health education about glaucoma.

NB: You do not have to agree to do these things, you don’t want to do**.** You can withdraw your consent at any time. All information that we collect will be confidential and no identifiable information will be released.

I acknowledge that I have understood this consent and the reasons for the study have been explained to me by my own language. I give my consent to participate in the study.

Respondent --------------------------------- sign and date---------------

Researcher/witness -----------------------sign and date ----------------
